# Characterization of the Poly-T Variant in the *TOMM40* Gene in Diverse Populations

**DOI:** 10.1371/journal.pone.0030994

**Published:** 2012-02-16

**Authors:** Colton Linnertz, Ann M. Saunders, Michael W. Lutz, Donna M. Crenshaw, Iris Grossman, Daniel K. Burns, Keith E. Whitfield, Michael A. Hauser, Jeanette J. McCarthy, Megan Ulmer, Rand Allingham, Kathleen A. Welsh-Bohmer, Allen D. Roses, Ornit Chiba-Falek

**Affiliations:** 1 Institute for Genome Sciences & Policy, Duke University, Durham, North Carolina, United States of America; 2 Deane Drug Discovery Institute, Duke University Medical Center, Durham, North Carolina, United States of America; 3 Division of Neurology, Department of Medicine, Duke University Medical Center, Durham, North Carolina, United States of America; 4 Cabernet Pharmaceuticals, Chapel Hill, North Carolina, United States of America; 5 Department of Psychology and Neuroscience, Duke University, Durham, North Carolina, United States of America; 6 Center for Human Genetics, Duke University Medical Center, Durham, North Carolina, United States of America; 7 Department of Ophthalmology, Duke University Medical Center, Durham, North Carolina, United States of America; 8 Joseph and Kathleen Bryan Alzheimer's Disease Research Center, Duke University, Durham, North Carolina, United States of America; 9 Department of Psychiatry and Behavioral Sciences, Duke University Medical Center, Durham, North Carolina, United States of America; University of Florida, United States of America

## Abstract

We previously discovered that a polymorphic, deoxythymidine-homopolymer (poly-T, rs10524523) in intron 6 of the *TOMM40* gene is associated with age-of-onset of Alzheimer's disease and with cognitive performance in elderly. Three allele groups were defined for rs10524523, hereafter ‘523’, based on the number of ‘T’-residues: ‘Short’ (S, T≤19), ‘Long’ (L, 20≤T≤29) and ‘Very Long’ (VL, T≥30). Homopolymers, particularly long homopolymers like ‘523’, are difficult to genotype because ‘slippage’ occurs during PCR-amplification. We initially genotyped this locus by PCR-amplification followed by Sanger-sequencing. However, we recognized the need to develop a higher-throughput genotyping method that is also accurate and reliable. Here we describe a new ‘523’ genotyping assay that is simple and inexpensive to perform in a standard molecular genetics laboratory. The assay is based on the detection of differences in PCR-fragment length using capillary electrophoresis. We discuss technical problems, solutions, and the steps taken for validation. We employed the novel assay to investigate the ‘523’ allele frequencies in different ethnicities. Whites and Hispanics have similar frequencies of S/L/VL alleles (0.45/0.11/0.44 and 0.43/0.09/0.48, respectively). In African-Americans, the frequency of the L-allele (0.10) is similar to Whites and Hispanics; however, the S-allele is more prevalent (0.65) and the VL-allele is concomitantly less frequent (0.25). The allele frequencies determined using the new methodology are compared to previous reports for Ghanaian, Japanese, Korean and Han Chinese cohorts. Finally, we studied the linkage pattern between *TOMM40*-‘523’ and *APOE* alleles. In Whites and Hispanics, consistent with previous reports, the L is primarily linked to ε4, while the majority of the VL and S are linked to ε3. Interestingly, in African-Americans, Ghanaians and Japanese, there is an increased frequency of the ‘523’S-*APOEε*4 haplotype. These data may be used as references for ‘523’ allele and ‘523’-*APOE* haplotype frequencies in diverse populations for the design of research studies and clinical trials.

## Introduction

Rs10524523 polymorphism, hereafter ‘523’, is a variable length, deoxythymidine homopolymer located in chromosome 19 at position 45403049 (Genome Build 37.1) within intron 6 of the *TOMM40* gene (Ensembl: ENSG00000130204). *TOMM40* encodes the essential mitochondrial protein import translocase (Translocase of the Outer Mitochondrial Membrane, 40 kD), and is adjacent to, and in linkage disequilibrium with, the apolipoprotein E (*APOE*) gene (Ensembl: ENSG00000130203). In the human reference sequence, the number of ‘T’ residues in the homopolymer is 35, and the variant allele described by rs10524523 is a 19 bp deletion (*i.e*. the variant allele is 16 T residues).

Using a deep sequencing and phylogenetic analysis approach, Roses *et al.* discovered that *TOMM40* ‘523’ contributes to the genetic risk and age of onset of late onset Alzheimer's disease (LOAD, MIM 104310) in *APOEε* 3/4 patients [1]. This polymorphism may explain some of the genetic association with age of disease onset previously attributed solely to *APOE* [1], [2]. A wide range of lengths are observed for this homopolymer (11–54 T), which may be binned into three major allelic groups according to the length distribution profiles: ‘Short’ (S, T≤19), ‘Long’ (L, 20≤T≤29) and ‘Very Long’ (VL, T≥30). [1], [2].

There is accumulating evidence that the poly-T locus in *TOMM40* is associated with progression to Alzheimer's disease (AD). The L and VL alleles are significantly associated with earlier age of disease onset in subjects carrying *APOE* ε3/4 [1], [2] (∼7 years earlier onset in VL/L vs. S/L) and *APOE* ε3/3 [3] (∼9 years earlier onset in VL/VL vs. S/S) genotypes. Johnson *et al.* described the association of VL alleles of ‘523’ with impaired verbal memory recall, known to be affected in the early stages of AD, in *APOE* ε3/3 subjects drawn from a clinically normal, late middle-aged cohort enriched for family history of AD [4]. Moreover, in a neuroimaging analysis of a subset of this APOE ε3/3 cohort, Johnson *et al.* demonstrated a significant association between ‘523’ VL and reduced gray matter volume, measured by MRI, in areas of the brain known to be affected in the early stages of AD [4]. More recently, an association between cognitive performance in cognitively normal elderly and ‘523’ genotype was identified; the ‘523’ variant was associated with neuropsychological measures that are preferentially affected in early stage AD (Hayden *et. al.* Alzheimer's and Dementia, *in press*).

Ongoing basic research into the biological significance of the ‘523’ poly-T locus and its potential for use in a clinical trial setting [5] inspired the need to develop a genotyping assay that is simpler and less expensive than the Sanger sequencing method that was used previously [1], [2], [4]. We have developed an assay that is sufficiently simple, inexpensive and robust to perform in a standard molecular genetics laboratory. We have used this assay to determine ‘523’ allele and ‘523’-*APOE* haplotype frequencies in diverse populations.

## Materials and Methods

### Subjects

#### Assay validation cohorts

DNA samples generously provided by the Kathleen Price Bryan Brain Bank (KPBBB) at Duke University. The assay was validated in three sample sets using a total of 140 DNA samples, 40 from brain tissue and 100 from blood. The Duke University Health System Institutional Review Board for Clinical Investigations granted approval for use of the Bryan ADRC Database/Repository.

#### Ethnic cohorts

White (of European descent): 177 individuals (∼70% females) from five local independent-living retirement communities in the Research Triangle Park region of North Carolina. The average age at recruitment was 80.4±6.1 (age range 63–95). DNA was extracted from saliva samples. This project was approved by the Duke Institutional Review Board.

African American: This group comprised 370 individuals (∼78% were females) sampled from 29 different independent housing buildings in Baltimore MD. The average age was 71.90±9.06 (age range 52–96). DNA was extracted from saliva samples. This project was approved by the Duke Institutional Review Board.

Hispanic: This group comprised 179 subjects (∼75% females) from Proyecto SALSA, a clinic-based sample of low-income Mexican Americans from San Diego County ascertained between 2003–2005. The average age of the adult Latino participants of that study was 54 years old. Blood samples were collected for DNA extraction. The project was approved by IRBs at San Diego State University and Duke University [6].

### DNA extraction

Saliva sample collection and DNA extraction were performed using the commercially available Oragene DNA Self-Collection Kit (DNA Genotek Inc., Kanata, Ontario, Canada) according to the manufacture's protocol. DNA extractions from blood and brain tissue were performed using the QIAamp DNA kit by the standard Qiagen protocol (Qiagen Valencia, CA, USA). DNA concentration and the quality of purification were determined spectrophotometrically.

### *TOMM40* poly-T rs10524523 genotyping assay

Each genomic DNA sample (10–20 ng) was PCR amplified using fluorescently labeled forward 5′FAM-TGCTGACCTCAAGCTGTCCTC-3′ and reverse 5′-GAGGCTGAGAAGGGAGGATT-3′primers (each 0.4 µM). The PCR amplification was carried out by TaKaRa EX Taq polymerase (Takara Bio Inc., Otsu, Shiga, Japan) in the presence of 5% fresh DMSO and under the following conditions: 3 min at 94°C, then 27 cycleswith 15 s at 94°C, 20 s at 65°C, and 30 s at 70°C; concluded with 5 min at 70°C. At completion, the reaction mix was maintained at 4°C. Whereas ‘N’ represents the number of the poly-T residues, the expected length of the PCR product is N+150 bp (the poly-T flanking region and an ‘A’ overhang at the end of the product).

Two µl of each PCR product, 7.5 µl of Hi-Di Formamide (Applied Biosystems, Foster City, CA) and 0.5 µl of Size Standard (GeneScan 500LIZ; Applied Biosystems, Foster City, CA) were denatured at 95°C for 3 min, immediately chilled in an ice water bath for 10 min, and loaded on an ABI 3730 DNA Analyzer. Genotypes were determined on an ABI 3730 using GeneMapper version 4.0 software (Applied Biosystems, Foster City, CA) for fragment analysis by the amplified fragment length polymorphism (AFLP) method. The ‘523’ alleles were called according to the length of the PCR product. The convention established by Roses *et al.* for determining alleles was used: Short (S), ≤19; Long (L) −20–29; Very Long (VL) ≥30 [1], [2].

### *APOE* genotyping

*APOE* genotypes were determined using a TaqMan-based allelic discrimination assay (Applied Biosystems). Briefly, *APOE* ε2/3/4 genotypes were established using two separate SNPs: (1) *rs429358* 334T/C (ABI assay ID: C_3084793_20), and (2) *rs7412* 472T/C (ABI assay ID: C_904973_10). The assays were conducted on a ABI 7900HT and genotype analysis was performed by the SNP auto-caller feature of SDS software. *APOE* genotypes were assigned as described previously [7].

## Results and Discussion

We have developed a genotyping assay that uses PCR amplification of the ‘523’ poly-T region followed by capillary electrophoresis of the PCR products to size the DNA fragments. The automated ABI 3730 sequencing platform yielded accurate sizing results with single-base resolution.

### Technical issues and solutions

Homopolymers and other Simple Polymeric Repeats (SPRs) are notoriously difficult to analyze, particularly with methods that employ PCR. The problem is that a significant amount of ‘slippage’ may occur during each DNA amplification cycle, causing the newly polymerized strand to have either fewer or more residues than the original template strand. After several cycles of PCR, the amplification product contains a complex mixture of PCR amplicons that vary in length at the poly-T locus and include amplicons with the true poly-T length. Each PCR sample, containing all amplicons, is analyzed by capillary electrophoresis to determine amplicon length. The polymerase ‘slippage’ is not completely avoidable, therefore the biggest hurdle to overcome with this assay is to find a way to analyze the complex electropherograms produced by capillary electrophoresis of the PCR products in order to determine precisely the original template length and thus the true poly-T length. To circumvent this problem, we took advantage of that fact that the lengths of the PCR amplicons are normally distributed (*i.e.* each electropherogram shows a cluster of peaks, due to slippage at the poly-T locus, with a normal distribution of peak heights). We assumed that the true amplicon length had the highest frequency in the mixture of PCR-product lengths, reflected by the highest intensity peak within the cluster (*i.e*. the fragment length of the highest peak, or the Mode value, indicates the original (pre-PCR) fragment length).

The automated analysis of the fragment lengths might introduce ±1 bp sizing differences in calling the absolute product length between runs. To overcome this issue we included consistent calibration standards in each run. These calibration samples, one for each ‘523’ allele category, were selected from a set of DNA samples with known poly-T lengths that were determined by direct Sanger sequencing (of multiple PCR clones). The calibration standards are included as parallel reactions subjected to the same PCR conditions and capillary electrophoresis, and used as reference controls for each run.

### Assay validation

Validation checks were performed in three stages: 1) using DNA from twelve subjects (extracted from 8 blood and 4 brain samples) an open comparison was made between the results of the new electrophoretic assay and the results of direct Sanger sequencing obtained by sequencing multiple clones of PCR products containing the ‘523’ poly-T (conducted by Polymorphic, Inc.) [1], [2]. The two methods agreed, for all allele categories (S/L/VL), with a maximum length deviation of ±1 bp. 2) 36 DNA samples extracted from brain tissue were used in a blinded comparison of the results of the new electrophoretic assay with the results of the Sanger sequencing-based assay (conducted by Polymorphic, Inc.). There was 100% concordance, as scored by a third party. 3) In a second blinded comparison we used 72 different DNA samples extracted from blood of subjects with the *APOE* ε3/4 genotype. DNA samples were genotyped for the ‘523’ polymorphism by the new electrophoretic assay and compared to the results obtained by the Sanger sequencing-based assay (conducted by Polymorphic, Inc.). In this test, there was 93% (67/72) concordance for ‘523’ genotype calls (using the S, L, VL categories) between the two methods. Numerical poly-T lengths were concordant 90% (129/144) of the time, where concordance was a difference of 0 or ±1 ‘T’ residue. The mean difference in allele length between the two assays was 1.1 (SD 1.9). It should be noted that both assays use PCR as a first step which may introduce variability that originated from ‘slippage’ as described above.

### Allele frequencies in diverse populations

The electrophoretic genotyping assay was employed to determine ‘523’ allele frequencies in diverse populations residing in the United States. Three races or ethnicities were investigated: Whites (individuals of European descent), African Americans, and Hispanics. Whites and Hispanics had similar allele distributions, which differed from the distribution in African Americans (Table 1). While the frequency of the L allele was similar across the different groups (9–11%), the frequencies of the S and VL alleles differed. In Whites and Hispanics, the S and VL alleles were common with similar frequencies (45% and 44%, respectively and 43% and 48%, respectively), but in African Americans the S allele was the most common (65%) and the VL allele was relatively less frequent (25%) (Table 1).

**Table 1:** ‘523’ Allele frequencies in different ethnicities in the US.

Ethnicity	Subjects(N)	S (%)	L(%)	VL(%)	Poly T length (range)
**Whites**	177	45	11	44	14–39
**African American**	370	65	10	25	14–54
**Hispanic**	179	43	9	48	14–39

The poly-T tracts ranged from 14 to 54 T residues (Table 1) taking into account all populations. Interestingly, the much longer homopolymers were noted in the African American group compared to the other groups studied (poly-T lengths up to 54 residues were seen in the African American group vs. 39 T residues in Whites and Hispanics, Table 1). These ’523’ allele distributions for the US populations were compared to previous results from Ghanaian, Japanese, Korean and Han Chinese cohorts(Table 2) [8]. ‘523’ allele frequencies for the Ghanaian cohort were most similar to the African American cohort, but there was an even greater enrichment for the S allele in the Ghanaian sample (71% for Ghanaian, 65% for African American). Interestingly, the Far Eastern cohorts (Japanese, Korean and Han Chinese) were enriched for the VL allele when compared to Whites and African Americans (52–72% for Far Eastern cohorts, 44% for Whites, 25% for African Americans) with a concomitant decrease in the frequency of the S allele (20–38% for the Far Eastern cohorts, 45% for Whites, 65% for African Americans).

**Table 2:** ‘523’ Allele frequencies in non US geographical cohorts (Far Eastern and West Africa).

Ethnicity	Subjects(N)	S (%)	L(%)	VL(%)	Poly T length (range)
**Ghanaian**	40	71	8	21	13–43
**Japanese**	60	24	18	58	11–35
**Korean**	60	20	8	72	11–38
**Han Chinese**	60	38	10	52	12–36

Genotypes determination was performed by Polymorphic, Inc. using a sequencing based assay.

*TOMM40* and *APOE*, are adjacent genes on chromosome 19 and are in high linkage disequilibrium (LD). We investigated the linkage between the ‘523’ alleles (S, L, VL) and the different alleles of *APOE* (ε2, ε3, or ε4). Table 3 presents the *APOE* allele frequencies for each ‘523’ genotype group. In Whites, the L allele most frequently co-occurs with ε4, while the majority of the VL and S alleles co-occur with ε3 (Table 3). This observation is consistent with the previous reports in White populations [1], [2]. A relatively similar frequency of *APOE*-‘523’ haplotypes was observed with the Hispanic population (Table 3). It is very important to note that, unlike the White populations (this study and previous reports) and the Hispanic population, the African Americans showed a significant number of ε4 alleles in the S/S subjects group (∼13% of the S alleles, Table 3). This result indicates that the S and the ε4 alleles were linked relatively frequently in the African American population. We also observed a relative higher frequency of ε4 allele in the VL containing genotypes in African-Americans compared to Whites and Hispanics (18% vs. 5%, 12%, respectively; Table 3), indicating that the ε4-VL haplotype is more frequent in the African Americans compared to the other populations. These ‘523’-*APOE* alleles haplotype frequencies for the US populations were compared to previous results from Ghanaian, Japanese, Korean and Han Chinese cohorts (Table 4) [8]. Consistent with the African Americans, the Ghanaian population also showed enrichment of ε4 alleles in S-containing genotypes (Table 4). Also, although the number of VL genotypes in the Ghanaian subjects was very small, the ε4-VL haplotype is likely at relatively higher frequency (Table 4). Among the Far Eastern cohorts the Korean and Han Chinese showed a similar allelic distribution to that observed in the White and Hispanic cohorts (Table 4). Interestingly, the haplotype data of the Japanese implied more frequent occurrence of *APOE* ε4-S haplotypes, similar to the observation with the African American and Ghanaian samples.

**Table 3:** *APOE* allele frequencies by ‘523’ genotypes in different ethnicities in the US.

523 genotype			S/S	L/L	VL/VL	S/L	S/VL	L/VL
**White**	*Subjects N*		*34*	*3*	*37*	*22*	*70*	*11*
	APOE	e4	0 (0)	6 (100)	2 (3)	21 (.48)	2 (1)	9 (41)
	alleles	e3	63 (93)	0 (0)	62 (84)	21 (.48)	126 (90)	13 (59)
	n (%)	e2	5 (7)	0 (0)	10 (13)	2 (4)	12 (9)	0 (0)
**African American**	*Subjects N*		*153*	*2*	*22*	*49*	*127*	*17*
	APOE	e4	38 (12)	4 (100)	6 (14)	52 (53)	35 (14)	18 (53)
	alleles	e3	225 (74)	0 (0)	37 (84)	39 (40)	202 (79)	16 (47)
	n (%)	e2	43 (14)	0 (0)	1 (2)	7 (7)	17 (7)	0 (0)
**Hispanic**	*Subjects N*		*34*	*3*	*39*	*10*	*77*	*16*
	APOE	e4	2 (3)	6 (100)	7 (9)	8 (40)	8 (5)	16 (50)
	alleles	e3	64 (94)	0 (0)	71 (91)	12 (60)	140 (91)	16 (50)
	n (%)	e2	2 (3)	0 (0)	0 (0)	0 (0)	6 (4)	0 (0)

**Table 4:** *APOE* allele frequencies by ‘523’ genotypes in non US geographical cohorts (Far Eastern and West Africa).

523 genotype			S/S	L/L	VL/VL	S/L	S/VL	L/VL
**Ghanaian**	*Subjects N*		*21*	*0*	*3*	*5*	*10*	*1*
	APOE	e4	3 (7)	0 (0)	1 (17)	4 (40)	5 (25)	2 (100)
	alleles	e3	28 (67)	0 (0)	4 (66)	5 (50)	10 (50)	0 (0)
	n (%)	e2	11 (26)	0 (0)	1 (17)	1 (10)	5 (25)	0 (0)
**Japanese**	*Subjects N*		*2*	*2*	*24*	*10*	*15*	*7*
	APOE	e4	2 (50)	4 (100)	0 (0)	6 (30)	3 (10)	3 (21)
	alleles	e3	1 (25)	0 (0)	48 (100)	13 (65)	26 (87)	11 (79)
	n (%)	e2	1 (25)	0 (0)	0 (0)	1 (5)	1 (3)	0 (0)
**Korean**	*Subjects N*		*2*	*0*	*30*	*2*	*18*	*8*
	APOE	e4	0 (0)	0 (0)	0 (0)	1 (25)	1 (3)	7 (44)
	alleles	e3	4 (100)	0 (0)	60 (100)	3 (75)	30 (83)	9 (56)
	n (%)	e2	0 (0)	0 (0)	0 (0)	0 (0)	5 (14)	0 (0)
**Han Chinese**	*Subjects N*		*7*	*1*	*15*	*4*	*27*	*6*
	APOE	e4	0 (0)	2 (100)	0 (0)	3 (37.5)	1 (2)	5 (42)
	alleles	e3	10 (71)	0 (0)	29 (97)	2 (.25)	46 (85)	7 (58)
	n (%)	e2	4 (29)	0 (0)	1 (3)	3 (37.5)	7 (13)	0 (0)

Genotypes determination was performed by Polymorphic, Inc.

The unique distribution of allele frequencies, differences in the poly-T lengths within each allele group, and the inferred differences in the linkage patterns between ‘523’ and *APOE* alleles emphasize the importance of extending this study to additional ethnic groups in the US and worldwide. It will be valuable to have direct measurement of the ‘523’-*APOE* haplotype frequencies in diverse groups.

The data will serve as standard references for ‘523’ allele frequencies in diverse populations for subsequent research studies, including investigations of age of onset of AD and/or cognitive decline in different ethnicities, and for future clinical trials.

### Associations of *TOMM40* ‘523’ with LOAD

Since Roses *et al*. discovered the association between ‘523’ and age of LOAD onset [1], [2], a number of studies have also examined this locus. Caselli *et al.* replicated the association between the longer ‘523’ alleles and earlier onset of LOAD in an independent group composed of *APOE ε*3/3 subjects drawn from a longitudinal study [3]. Furthermore, Johnson et al. reported significant association of ‘523’ with impaired cognition and brain atrophy in a clinically normal, late middle-aged cohort of *APOE* ε3/3 subjects drawn from a population enriched for family history of LOAD. In this cohort, the ‘523’ VL allele was significantly associated with worse performance on primacy retrieval from a verbal list learning task and with reduced gray matter volume in ventral posterior cingulate and medial ventral precuneus, both known to be affected in early AD [4]. In a cross sectional study of cognitively healthy elderly, we have also observed *APOE*-independent associations between the ‘523’ polymorphism and specific cognitive domains of memory and executive control that are preferentially affected in early stage AD (Hayden *et. al.* Alzheimer's and Dementia, *in press*). Together with the original findings, these new studies establish, in Whites, the importance of ‘523’ in AD pathogenesis, particularly for *APOE* ε3 carriers. However, there are conflicting reports. While Chu *et al.* did not replicate the association between ‘523’ and age of AD onset [9], Cruchaga *et al.* replicated the association between the ‘523’ and AD in *APOE* ε3/3 subjects, but found that the S allele, rather than the VL allele, was associated with increased AD risk; *i.e. APOE* ε3/3 subjects who carried the S/S genotype showed a trend towards earlier age of onset [10]. Maruszak *et. al*. observed a significant association between ‘523’ and LOAD risk, but reported that the ε3-VL haplotype is significantly more frequent among patients with a later age of onset (≥79 years) contrary to the prediction of the original report [11]. Roses commented on these conflicting observations and has proposed that there is a further subdivision of VL subjects, with one VL subgroup falling prey to very early onset disease ([12] and unpublished data). Some of the discrepancies between the studies might be explained by the method used to ascertain the age of onset. Different results may be attributed to issues associated with study design, *e.g.* prospective versus retrospective, longitudinal versus cross-sectional [1], [2] [3] [10], [11] [12]. The differences in the results could also be related to technical limitations and quality control of the ‘523’ genotyping assay. Well-powered, longitudinal studies in diverse populations with careful determination of age of onset using established criteria and accurate genotypes determined by a validated method are needed. This paper presents one part of the solution – a validated, high-throughput assay for accurate determination of ‘523’ allele length.
